# Acute Phase Response in Patients With Uncomplicated and Complicated Endoscopic Retrogradic Cholangiopancreaticography

**DOI:** 10.1155/1994/69467

**Published:** 1994

**Authors:** Heikki Kiviniemi, Tatu Juvonen, Jyrki Mäkelä

**Affiliations:** Department of Surgery Oulu University Hospital Oulu FIN-90220 Finland

## Abstract

Acute phase response after endoscopic retrogradic cholangiopancreaticography (ERCP) was studied in 42 patients with suspected pancreatic or biliary diseases. In uncomplicated cases acute phase response determined by serum C-reactive protein levels was rare and did not parallel the serum amylase or lipase levels. In three of the these 42 patients, post-ERCP pancreatitis developed and CRP levels elevated sharply and paralleled the degree of pancreatitis. Six additional patients outside of this prospective study with post-ERCP-pancreatitis and daily CRP determinations were used to determine the CRP-response curve in post-ERCP pancreatitis.


*HPB Surgery*, 1994, Vol. 8, pp. 129–131
Reprints available directly from the publisher
Photocopying permitted by license only

© 1994 Harwood Academic Publishers GmbH
Printed in Malaysia

SHORT COMMUNICATION

### Acute Phase Response in Patients with Uncomplicated and Complicated Endoscopic Retrogradic Cholangiopancreaticography

HEIKKI KIVINIEMI, TATU JUVONEN and JYRKI MÄKELÄ

Department of Surgery, Oulu University Hospital, FIN-90220 Oulu, Finland

Acute phase response after endoscopic retrogradic cholangiopancreaticography (ERCP) was studied in 42 patients with suspected pancreatic or biliary diseases. In uncomplicated cases acute phase response determined by serum C-reactive protein levels was rare and did not parallel the serum amylase or lipase levels. In three of the these 42 patients, post-ERCP pancreatitis developed and CRP levels elevated sharply and paralleled the degree of pancreatitis. Six additional patients outside of this prospective study with post-ERCP-pancreatitis and daily CRP determinations were used to determine the CRP-response curve in post-ERCP pancreatitis.

### INTRODUCTION

The clinical symptoms after endoscopic retrogradic cholangiopancreaticography (ERCP) are usually moderate, but acute pancreatitis has been described in 1–7 %[1-3]. Hyperamylasemia following ERCP, as a sign of pancreatic injury, occurs in up to 70% [4,5] after endoscopic pancreaticography (ERP). Lipase has been suggested to be an even more sensitive indicator of pancreatic injury than amylase[6]. According to recent reports C-reactive protein (CRP), an acute phase protein secreted by hepatocytes—is valuable in estimation of tissue injury[7] and infectious complications[8]. Our purpose was to study CRP response in uncomplicated and complicated ERCP.

### MATERIALS AND METHODS

ERCP was performed in 42 consecutive patients with suspected pancreatic or biliary diseases, in a prospective manner. Before the examination the patients received sedation and anticholinergic agent (Buscopan[R]) to reduce gut mobility during the study. The blood samples were drawn for estimation of amylase, lipase and CRP values before cannulation of the papilla of Vater ($n$ = 42), six hours after ($n$ = 42), twenty-four hours after ($n$ = 42) cannulation.

The serum amylase and lipase values were determined automatically. CRP was measured using an immunoturbidic method (9), with reference < 12 mg per liter.

The patients were discharged from the hospital 24 hours after ERCP if no complications occurred. In cases of suspected acute pancreatitis amylase, lipase and CRP values were determined daily. Six additional patients with post-ERCP pancreatitis with daily CRP determinations were included in the study in order to formulate CRP response curve in post-ERCP pancreatitis. These patients did not belong to the prospective series of 42 patients, being treated before the beginning of this prospective series.

### RESULTS

Visualization of the pancreatic duct only (ERP) was successful in 22 cases, both the pancreatic duct and bile duct (ERCP) in 16 cases and the bile duct only in 4 cases.

*Address for correspondence*: Tatu Juvonen, Department of Surgery, Oulu University Hospital, FIN–90220 Oulu Finland Tel: + 358 81 315 2011, Fax: + 358 81 315 5318

**Table I** Serum C–reactive protein (CRP), lipase and amylase levels after ERCP in 42 patients

| *C–reactive protein (mg/l)* | *6 hours* | *24 hours* |
|---|---|---|
| < 13 | 39 | 39 |
| 13–20 | 3* | 2* |
| > 20 | 0 | 1* |
| Lipase (U/l) | | |
| < 160 | 16 | 21 |
| 160–300 | 9 | 8 |
| > 300 | 17* | 13* |
| Amylase (U/l) | | |
| < 300 | 20 | 25 |
| 300–600 | 13 | 6 |
| > 600 | 9* | 11* |

* Three patients with post ERCP pancreatitis are included in the group.

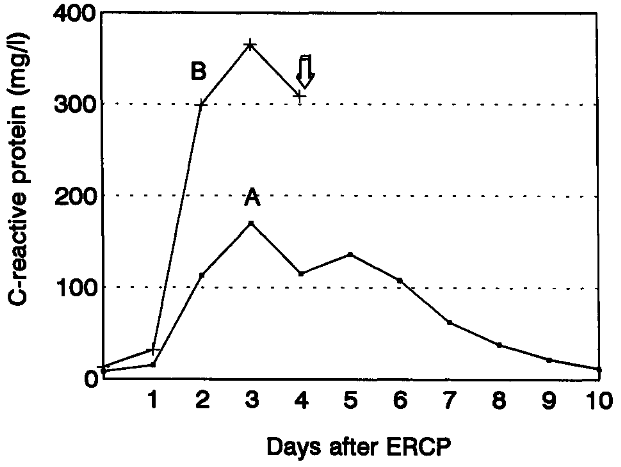

**Figure 2** C-reactive protein response in a patient with post-ERCP pancreatitis (A) and acute haemorrhagic pancreatitis (B). The arrow indicates the time of surgical intervention.

The behaviour of CRP, amylase and lipase did not differ between the groups and are presented in Table I. In uncomplicated cases lipase is most sensitive, whereas CRP elevation above the reference range was rare. In 5 patients with uncomplicated ERCP CRP values stayed normal for up to forty-eight hours of the study (data not shown). In 3 of the 42 prospective patients acute pancreatitis (AP) developed, lipase and amylase was raised markedly in these patients, a strong CRP response was also seen on the second day after the cannulation of the papilla of Vater.

In addition to these 3 patients with AP, 6 additional patients with post-ERCP pancreatitis with daily CRP determinations were studied retrospectively. The CRP response in these 9 patients with post-ERCP pancreatitis is seen in Figure 1. Two of these patients had a fulminant form of acute pancreatitis and were operated on. Debridement was performed in these patients. There was no mortality. A typical CRP-response curve in nonhaemorrhagic and fulminant case, respectively, is presented in Figure 2.

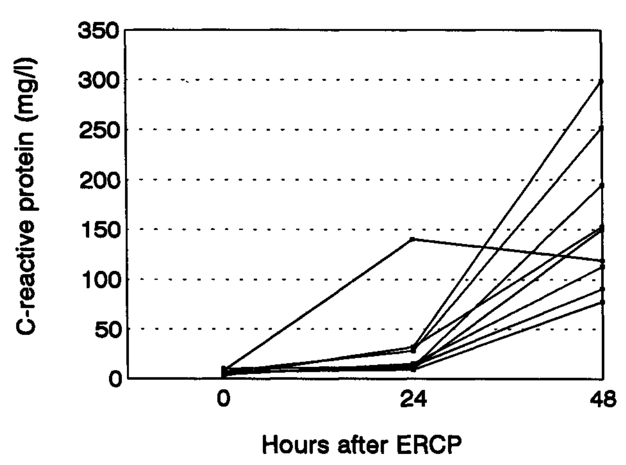

**Figure 1** C-reactive protein response in 9 patients with post-ERCP pancreatitis.

### DISCUSSION

The development of post-ERCP pancreatitis is a serious complication associated with fulminant forms of the disease[10] and also with mortality[11]. CRP is helpful in monitoring and assessing the severity of acute pancreatitis[12]. When the patients are admitted to hospital with acute pancreatitis, they have variable symptoms. Often the time of onset of non-ERCP-pancreatitis cannot be estimated exactly and CRP response is variable (13). In post-ERCP pancreatitis the time of cannulation and injection of contrast medium is known and CRP response curve can be estimated accurately. In all our patients, the initial CRP values were normal.

In accordance with other studies (6) serum lipase and amylase were elevated very early in over 50% of uncomplicated ERCP cases, whereas CRP reponse could be seen very rarely. So, CRP seems not to be a sensitive indicator of enzyme release.

In cases of post-ERCP pancreatitis CRP the response was substantial, showing highly elevated values at 48 hours after ERCP (mostly over 100 mg/l). The delay in this response depends on mediators, such as interleukin-1, which are essential to the acute phase response[14]. Many patients with post-ERCP pancreatitis get necrosis[15] and pain is a most important sign of this developing complication. Two of our nine post-ERCP pancreatitis patients were severe (22 %). According to recent literature severe pancreatitis after ERCP develops in 16 % of cases[15].

The determination of CRP response in suspected post-ERCP pancreatitis is obviously useful, because it reflects-in contrast to amylase and lipase—the degree of pancreatic injury and the course of the disease episode.

### REFERENCES

1. Bilbao, M. K., Dotter, C. T., Lee, T. G. *et al.* (1976) Complications of endoscopic retrograde cholangiopancreatiography (ERCP). *Gastroenterology* **70**, 314–318.
2. Koch, H., Belohlavek, D., Schaffner, O. *et al.* (1975) Prospective study for the prevention of pancreatitis following endoscopic retrograde cholangiopancreaticography (ERCP). *Endoscopy* **7**, 221–228.
3. Nebel, O. T., Silvis, S. E., Rogers, G. *et al.* (1975) Complications associated with endoscopic retrograde cholangiopancreaticography. *Gastrointest Endoscopy* **22**, 34–38.
4. Cotton, P. B., (1972) Cannulation of the papilla of Vater by endoscopy and retrograde cholangiopancreatography (ERCP). *Gut* **13**, 1014–1016.
5. La Ferla, G., Gordon, S., Archibald, R. G. N. *et al.* (1986) Hyperamylasemic and acute pancreatitis following endoscopic retrograde cholangiopancreatography. *Pancreas* **1**, 160–163.
6. Fjosne, U., Waldum, H. L., Romslo, I. *et al.* (1986) Amylase, pancreatic isoamylase and lipase in serum before and after endoscopic pancreatiography. *Acta. Med. Scand.* **219**, 301–304.
7. Gewarz, H., Carolyn, M., Siegel, J. *et al.* (1982) C-reactive protein and the acute phase response. *Adv. Intern. Med.* **27**, 345–372.
8. Schentag, II., O'Keefe, D., Marmion, M. *et al.* (1984) C-reactive protein as an indicator of infection relapse in patients with abdominal sepsis. *Arch Surg* **119**, 300–304.
9. Harmoinen, A., Perko, M., Grönroos, P. (1981) Rapid quantitative determination of C-reactive protein using LKB 8600 Reaction Rate Analyzer. *Clin. Chim. Acta.* **111**, 117–120.
10. Seifert, E., Gail, K., Weissmüller, J. (1982) Langzeitresultate nach endoscopischer Sphinkterotomie. Follow-up Studie aus 25 Zentren in der Bundesrepublik. *Deutsch Med Wochenschr* **107**, 610.
11. Stanton, F., Frey, C. F., (1990) Pancreatitis after endoscopic retrograde cholangiopancreaticography. *Arch. Surg.* **125**, 1032–1035.
12. Mayer, A. D., McMahon, M. J., Bowen, M. *et al.* (1984) C-reactive protein: an aid to assessment and monitoring of acute pancreatitis. *J. Clin. Pathol.* **37**, 207–211.
13. Puolakkainen, P., Valtonen, V., Paananen, A. *et al.* (1987) C-reactive protein (CRP) and serum phospholipase $A_2$ in the assessment of the severity of acute pancreatitis. *Gut.* **28**, 764–774.
14. Dinarella, C. A., (1984) Interleukin-1 and the pathogenesis of the acutephase response. *N. Engl. J. Med.* **311**, 1413–1418.
15. Nordback, I., Airo, I., (1988) Post-ERCP necrotizing pancreatitis. *Ann. Chir. Gyn.* **77**, 15–20.

